# The Cytoplasmic Capping Complex Assembles on Adapter Protein Nck1 Bound to the Proline-Rich C-Terminus of Mammalian Capping Enzyme

**DOI:** 10.1371/journal.pbio.1001933

**Published:** 2014-08-19

**Authors:** Chandrama Mukherjee, Baskar Bakthavachalu, Daniel R. Schoenberg

**Affiliations:** 1 Center for RNA Biology, The Ohio State University, Columbus, Ohio, United States of America; 2 Department of Molecular & Cellular Biochemistry, The Ohio State University, Columbus, Ohio, United States of America; Max Planck Institute for Developmental Biology, Germany

## Abstract

Cytoplasmic capping is catalyzed by a complex that contains capping enzyme (CE) and a kinase that converts RNA with a 5′-monophosphate end to a 5′ diphosphate for subsequent addition of guanylic acid (GMP). We identify the proline-rich C-terminus as a new domain of CE that is required for its participation in cytoplasmic capping, and show the cytoplasmic capping complex assembles on Nck1, an adapter protein with functions in translation and tyrosine kinase signaling. Binding is specific to Nck1 and is independent of RNA. We show by sedimentation and gel filtration that Nck1 and CE are together in a larger complex, that the complex can assemble *in vitro* on recombinant Nck1, and Nck1 knockdown disrupts the integrity of the complex. CE and the 5′ kinase are juxtaposed by binding to the adjacent domains of Nck1, and cap homeostasis is inhibited by Nck1 with inactivating mutations in each of these domains. These results identify a new domain of CE that is specific to its function in cytoplasmic capping, and a new role for Nck1 in regulating gene expression through its role as the scaffold for assembly of the cytoplasmic capping complex.

## Abstract 2

mRNA capping and decapping requires a cytoplasmic complex to maintain and/or restore the 5′ cap on a subset of the mammalian transcriptome; Nck1, an SH2/SH3 adapter, creates a scaffold upon which the cytoplasmic capping complex forms.

## Author Summary

We previously described a cyclical process of mRNA decapping and recapping termed “cap homeostasis.” Recapping is catalyzed by a complex of cytoplasmic proteins that includes the enzyme known to catalyze nuclear capping, and a kinase that converts RNA with a 5′-monophosphate end to a 5′-diphosphate capping substrate. The current study shows these two enzymatic activities are brought together in the cytoplasmic capping complex as both bind to adjacent domains of the adapter protein Nck1. Nck1 is a cytoplasmic protein best known for transducing receptor tyrosine kinase signaling. We identify a proline-rich sequence at the C-terminus of a human capping enzyme that is required for binding to Nck1, and we show that this interaction is required for integrity of the cytoplasmic capping complex. Depletion of Nck1 causes the cytoplasmic capping complex to dissociate. The inhibition of cytoplasmic capping by Nck1 with mutations in either the 5′-kinase or capping enzyme binding sites identified a functional role for Nck1 in cap homeostasis and a previously unknown function for Nck1 in cell biology.

## Introduction

The 7-methylguanosine “cap” is a defining feature of all eukaryotic mRNAs, and the cap plays a role in almost every step of mRNA metabolism. In the nucleus, the cap is bound by a heterodimer of CBP80-CBP20, and its interaction with other proteins coordinates many of the subsequent steps in pre-mRNA processing and mRNA surveillance [1]. mRNAs are exported to the cytoplasm cap-end first, where the CBP80-CBP20 heterodimer is replaced by eIF4E, leading to translation initiation through the eIF4F complex. Translation and mRNA decay are interconnected processes, and for many transcripts loss of the cap is thought to be an irreversible step leading to mRNA decay [2].

Nuclear capping is catalyzed by the sequential actions of capping enzyme (RNGTT, RNA guanylyltransferase, and 5′-phosphatase, CE) and RNA cap methyltransferase (RNMT), both of which are positioned at the transcription start site by their binding to the C-terminal domain of RNA polymerase II [3]. A number of approaches use the cap to map transcription start sites. These include paired end analysis of transcription start sites (PEAT) [4], Capped analysis of gene expression (CAGE) [5], and RNA annotation and mapping of promoters for the analysis of gene expression (RAMPAGE) [6]. In human cells ∼72% of transcription start sites have matching CAGE data [7]. However, a significant number of CAGE tags do not correspond to transcription start sites [8], mapping instead to locations within the body of the transcript. Intriguingly, there is no evidence for downstream CAGE tags in the *Drosophila* transcriptome [6], suggesting that the presence of capped ends located downstream within the transcript body is unique to higher metazoans.

The decay of nonsense-containing human β-globin mRNA in erythroid cells results in the accumulation of a reproducible pattern of metastable decay intermediates that are missing sequences of their 5′ ends [9]. These were previously characterized as having a cap or cap-like structure on their 5′ ends [10], and it was in the course of re-examining this observation that we discovered cytoplasmic capping [11]. In nuclear capping, the diphosphate substrate for guanylic acid (GMP) addition is generated by hydrolysis of the β-γ phosphate bond on the 5′ ends of newly transcribed pre-mRNA, followed by the transfer of GMP bound covalently at lysine 294 to generate GpppX, where X is the 5′-most nucleotide. In cytoplasmic capping the proximal substrate for cytoplasmic capping is also a 5′-diphosphate, but this is generated by a 5′-monophosphate kinase that sediments with CE in a ∼140 kDa complex [11].

Our first *in vivo* experiments looked at the impact of inhibiting cytoplasmic capping on cellular recovery from stress. Stress was selected for study because it results in a generalized inhibition of translation, with non-translating mRNPs accumulating in P bodies and stress granules [12]. We reasoned that some transcripts might be stored in an uncapped state, and cytoplasmic capping might be required to restore these to the translating pool. Support for this hypothesis was seen in Otsuka and colleagues [11], where the ability of cells to recover from a brief arsenite stress was reduced by expression of an inactive form of capping enzyme (termed K294A) that is restricted to the cytoplasm by deletion of the nuclear localization sequence and addition of the HIV Rev nuclear export sequence. Proof that K294A expression inhibits cytoplasmic capping came from work in Mukherjee and colleagues [13]. The original purpose of that study was to identify mRNAs that are regulated by cytoplasmic capping, and in the course of doing so we discovered a cyclical process of decapping and recapping that we termed “cap homeostasis.” Cytoplasmic capping targets can be grouped into three categories on the basis of their cap status and stability in cells that are inhibited for cytoplasmic capping. One group of natively uncapped transcripts is destabilized when cytoplasmic capping is inhibited. In Mukherjee and colleagues [13] these are referred to as the “uninduced” pool. Another group has natively uncapped transcripts that are not destabilized. Instead, inhibition of cytoplasmic capping results in an increase in the uncapped population of each of the “common” transcripts. The third group accumulates uncapped forms only when cytoplasmic capping is inhibited. There is no change in the steady-state level of these “capping inhibited” mRNAs, and their uncapped forms accumulate in non-translating mRNPs. A number of these transcripts encode proteins that are involved in the mitotic cycle, which may explain the reduced survival of K294A-expressing cells after arsenite stress.

Ultimately, progress in understanding the function of cytoplasmic capping depends on identifying the components of the cytoplasmic capping complex and determining how the CE, the 5′-kinase that generates the diphosphate substrate, and a cap methyltransferase are brought together in a single complex. We noticed that modifications to the C-terminus reduced the relative amount of kinase and capping activity recovered from cytoplasmic extracts with CE. Because these modifications had no impact on covalent binding of GMP (i.e., guanylylation activity), this suggested the C-terminus might play a role in assembling the cytoplasmic capping complex. A search for functional domains identified a proline-rich SH3 binding site close to the C-terminus of vertebrate CE, but not in CE of lower metazoans. *Drosophila* CE has a run of three prolines, but there is an additional 34 amino acids that separates these from the C-terminus. The current study identifies this region as a third domain of CE that is bound by adapter protein Nck1, which in turn brings CE together with the 5′-monophosphate kinase to form the core of the cytoplasmic capping complex.

## Results

### C-Terminus of Capping Enzyme Is Required for Cytoplasmic Capping

**Figure 1:**
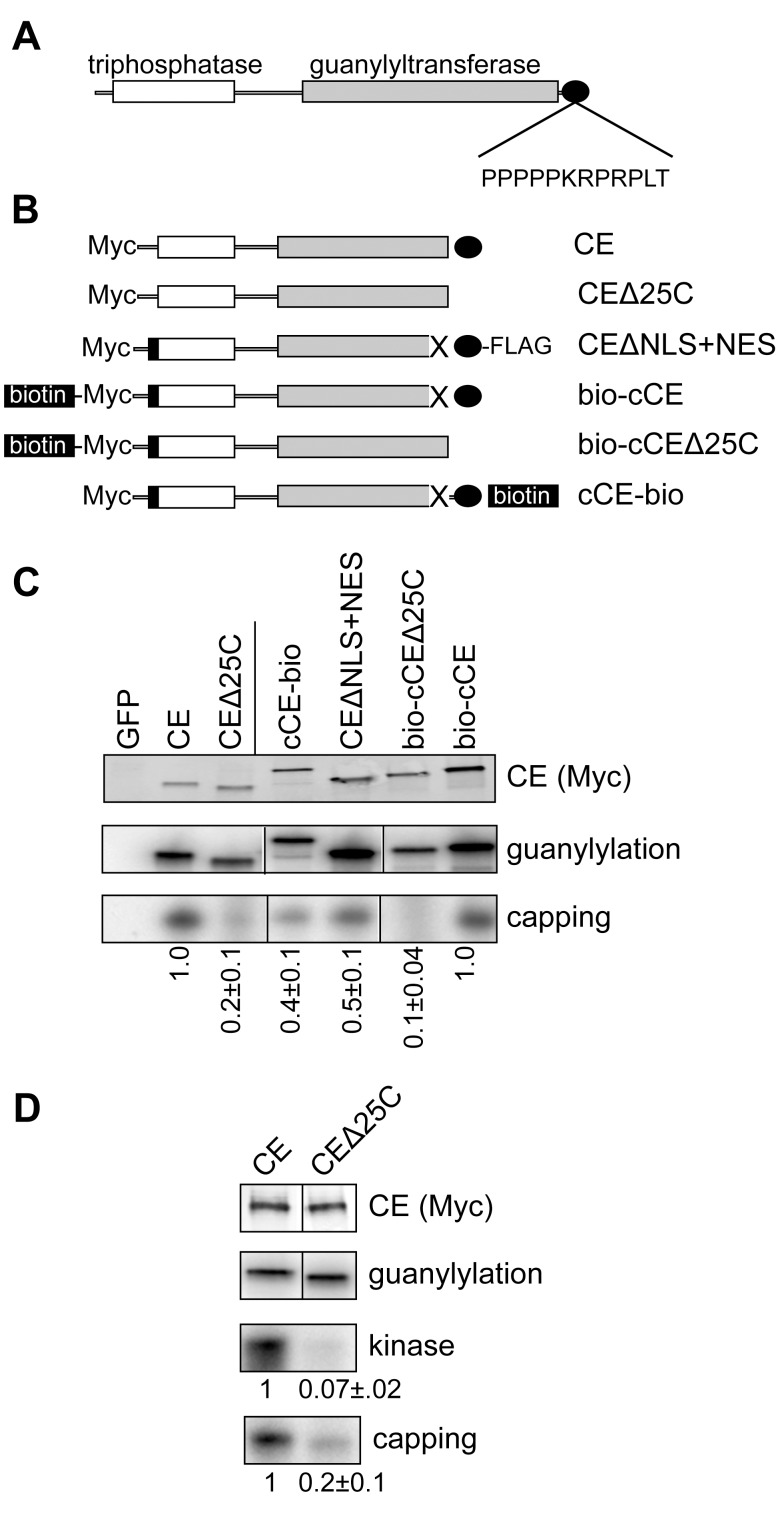
The capping enzyme C-terminus participates in assembly of the cytoplasmic capping complex. (A) The organization of vertebrate CE is shown with the N-terminal triphosphatase domain indicated in white, the guanylyltransferase domain indicated in grey, and the proline-rich C-terminus indicated by the black oval. The proximity of the proline-rich sequence to the C-terminus is indicated by the sequence of the last 12 amino acids of human CE. (B) The forms of CE used to analyze the impact of C-terminal modifications is shown. In the nomenclature used here CE corresponds to full-length protein with an N-terminal Myc tag. CEΔ25C is the same protein missing the C-terminal 25 amino acids. This deletion includes the NLS. CEΔNLS+NES was described in Otsuka and colleagues [11] and consists of Myc-tagged CE with the NLS deleted (X), a C-terminal FLAG tag and the HIV Rev NES (black box). In bio-cCE the NLS is deleted and a sequence that is biotinylated *in vivo* is added upstream of Myc tag and Rev NES. In bio-cCEΔ25C the C-terminal 25 amino acids of this protein were deleted. cCE-bio is similar to CEΔNLS+NES except that the C-terminal FLAG tag is replaced with the biotinylation sequence. (C) The plasmids shown in (B) or a plasmid expressing Myc-tagged GFP were transfected into HEK293 cells. GFP or CE and its associated proteins were recovered with anti-Myc beads (CE, CEΔ25C, cCE-bio, and CEΔNLS+NES) or streptavidin beads (bio-cCE and bio-cCEΔ25C). The recovered protein was incubated with α-[^32^P]GTP and analyzed for the covalent binding of [^32^P]GMP (guanylylation) [11], and for [^32^P]GMP labeling of 5′-monophosphate RNA (capping). The products were separated by denaturing gel electrophoresis and visualized by autoradiography. The amount of capping activity relative to guanylylation activity is shown at the bottom of the figure, with results normalized to either CE or to bio-cCE as indicated by the vertical line. (D) The experiment in (C) was repeated with an additional assay for 5′-kinase activity. The vertical line in the Western blot and guanylylation assay is to indicate that CE and CEΔ25C were separated by empty lanes on each of these gels. In (C and D) the mean ± standard deviation (*n* = 3) for recovered kinase and capping activity normalized to guanylylation activity is shown beneath each autoradiogram. In each case comparison to matching controls yielded *p*-value<0.05 by Student's *t* test.

Murine and human CE have proline-rich sequences immediately upstream of their C-termini (Figure 1A), whereas the three prolines in *Drosophila* CE are separated from the C-terminus by 34 amino acids (Figure S1). To determine if differences here are relevant for cytoplasmic capping we examined the impact of modifying this portion of the protein (Figure 1C) on the *in vitro* activity of the cytoplasmic capping complex recovered from cells that were transfected with the constructs shown in Figure 1B. The proteins analyzed here included wild-type enzyme (CE), the same protein missing 25 amino acids from the C-terminus (CEΔ25C), the cytoplasmically restricted form of CE described in [11] (CEΔNLS+NES), which has an N-terminal Myc tag and a C-terminal FLAG tag, a similar construct without FLAG that has an added N-terminal sequence that becomes biotinylated *in vivo* (bio-cCE), the same construct missing the C-terminal 25 amino acids (bio-cCEΔ25C), and a construct similar to CEΔNLS+NES in which the C-terminal FLAG tag was replaced with one that is biotinylated (cCE-bio). These plasmids or a control expressing Myc-GFP were transfected into 293 cells and cytoplasmic forms of each of the epitope-tagged proteins and their associated partners was recovered using anti-Myc or streptavidin paramagnetic beads (Figure 1C, upper panel).

The first experiments examined the impact of the C-terminal modifications on covalent binding of GMP (guanylylation) to the active site lysine at 294 [14]. Proteins recovered from transfected cells were incubated with α-[^32^P]GTP and analyzed by SDS-PAGE and autoradiography (Figure 1C, middle panel). For the most part the amount of [^32^P]GMP bound covalently to each of these proteins matched the relative amount of protein determined by Western blotting (Figure 1C, upper panel), indicating the C-terminal modifications had little or no impact on the ability of these proteins to bind GMP. In Otsuka and colleagues [11] we described a functional *in vitro* capping assay that measures the labeling of a 23 nt long 5′-monophosphate RNA with α-[^32^P]GTP in a reaction containing unlabeled ATP. While the C-terminal modifications had relatively little impact on guanylylation activity they each reduced *in vitro* capping activity of the recovered proteins (Figure 1C, bottom panel).

The *in vitro* capping assay depends on the activity of a 5′-kinase to generate a diphosphate substrate for transfer of GMP [11]. The recovery of this activity with the different forms of CE was examined by incubating the recovered proteins and 5′-monophosphate RNA with γ-[^32^P]ATP (Figures 1D and S2). Again the Δ25C deletion had no impact on recovery of CE or guanylylation of the recovered protein; however, it resulted in the parallel loss of kinase and capping activities. The experiment in Figure S2 also included a [^32^P]labeled, capped human β-globin transcript that was added to each reaction to control for contaminating ribonuclease activity. The similar recovery of this RNA from each of the reactions confirmed that the differences seen with each of the C-terminal modifications were due to differences in activity of the complex.

### Nck1 Binds to the Proline-Rich C-Terminus of Cytoplasmic CE

**Figure 2:**
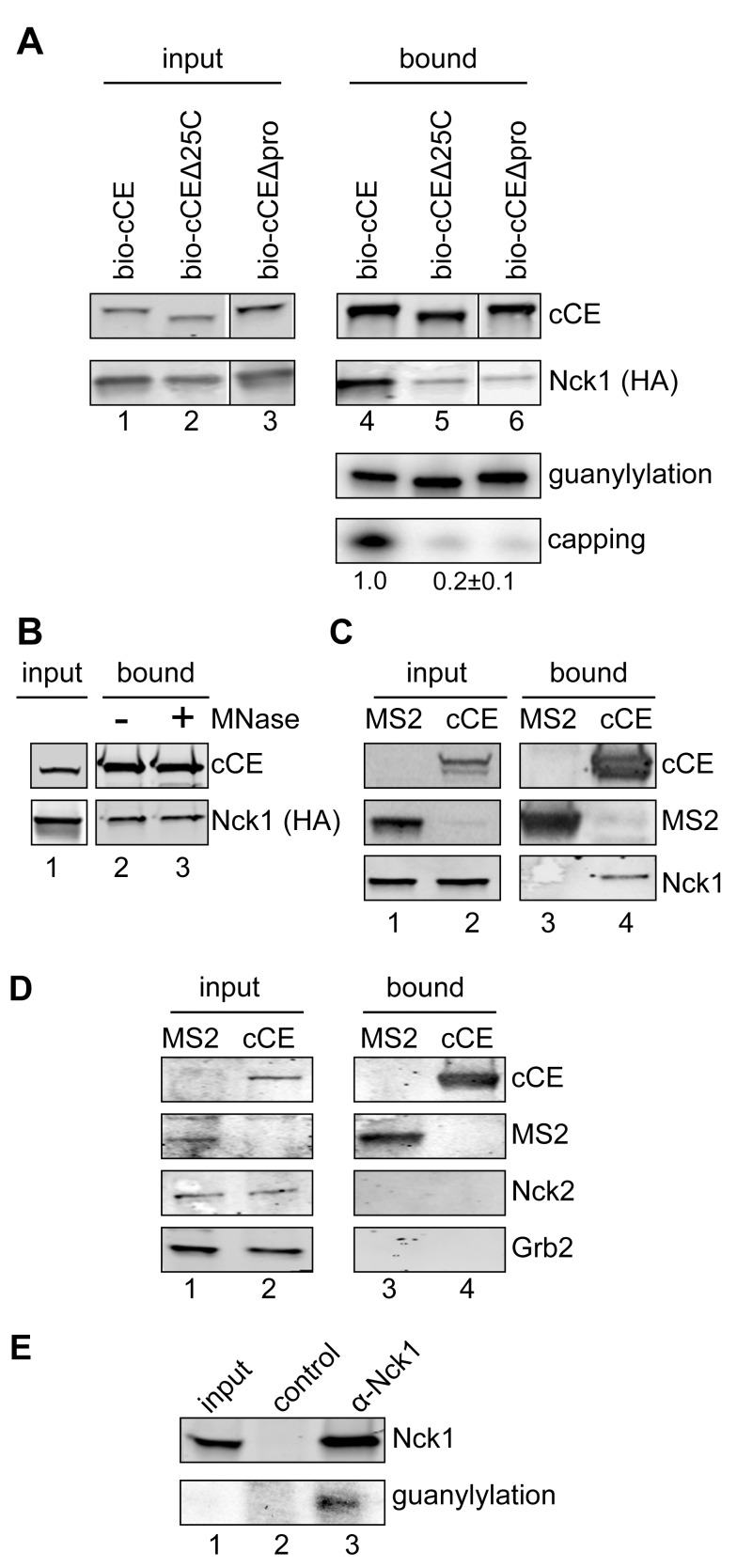
Identification of Nck1 binding to cytoplasmic CE. (A) HEK293 cells were co-transfected with plasmids expressing HA-Nck1 and bio-cCE (lanes 1 and 4), bio-cCEΔ25C (lanes 2 and 5), and bio-cCEΔpro in which the five C-terminal prolines shown in Figure 1A were deleted (bio-cCEΔpro, lanes 3 and 6). Protein recovered on streptavidin beads was analyzed by Western blotting with anti-Myc (cCE) and anti-HA (Nck1) antibodies (upper two panels). In the lower two panels the bound complexes were assayed as in Figure 1 for guanylylation and capping activity. The quantified capping activity (mean ± standard deviation, *n* = 3) for capping assay is shown beneath that autoradiogram. The same results were obtained for each of the modified forms of CE, with *p*-value<0.05 by Student's *t* test. (B) Cytoplasmic extract from cells expressing bio-cCE (cCE) and HA-Nck1 was treated ± micrococcal nuclease (MNase) prior to recovery of CE and associated proteins with streptavidin beads. Proteins were analyzed by Western blotting with anti-Myc (cCE) and anti-HA (Nck1) antibodies. (C) HEK293 cells were transfected with plasmids expressing bio-cCE or a protein consisting of two MS2 binding sites fused to the biotinylation sequence present in bio-cCE (MS2-bio) [19]. Proteins recovered on streptavidin beads were analyzed by Western blotting with HRP-streptavidin (bio-cCE and MS2-bio), and with an antibody to endogenous Nck1. (D) The experiment in (C) was repeated except Western blots of recovered proteins were probed with antibodies to Nck2 and Grb2. (E) Cytoplasmic extract was immunoprecipitated with control IgG or anti-Nck1 antibody. 2.5% of input sample and 20% of the immunoprecipitated sample was used for Western blotting to determine Nck1 recovery, and 2.5% of input sample and 70% of the immunoprecipitated sample was assayed by guanylylation to determine CE recovery. The weak guanylylation signal seen in the input sample is due to the presence of a previously described [11] inhibitory activity in cytoplasmic extract.

MIT ScanSite [15] identified adapter protein Nck1 (NP_006144.1) as a potential binding partner for the proline-rich C-terminus. Nck1 has 3 SH3 domains and a single C-terminal SH2 domain, and it has roles in transducing tyrosine kinase signaling [16], in translation [17] and in development [18]. It is classified as a cytoplasmic protein, and this was confirmed by Western blotting of nuclear and cytoplasmic extracts and by indirect immunofluorescence (Figure S3). To determine if Nck1 binds the proline-rich C-terminus we examined its recovery with bio-cCE, bio-cCE missing the C-terminal 25 amino acids (bio-cCEΔ25C), or bio-cCE missing the five C-terminal proline residues (bio-cCEΔpro). Cytoplasmic extracts from cells that were co-transfected with plasmids expressing each of these forms of CE and HA-Nck1 were recovered on streptavidin beads and assayed for Nck1, guanylylation, and capping activities (Figure 2A). As in the preceding experiment loss of the proline-rich C-terminus sequence had no impact on guanylylation activity. However, each of the deletions affected the recovery of both Nck1 and *in vitro* capping activity. The recovery of Nck1 with cCE was unaffected by prior treatment with micrococcal nuclease (Figure 2B), indicating RNA is not required for the interaction between these proteins. The binding of HA-Nck1 to CE is also independent of GMP binding as changing the active site lysine to alanine (K294A) had no effect on its recovery (Figure S4).

We next examined if endogenous Nck1 also binds to cytoplasmic CE. In Figure 2C cells were transfected with plasmids expressing bio-cCE or a protein with two copies of MS2 binding protein fused to the same biotinylated peptide sequence [19]. Selective binding of cCE by endogenous Nck1 was confirmed by Western blotting of protein recovered on streptavidin beads with anti-Nck1. Most cells also express Nck2 (NCK adapter protein 2, NP_001004720.1) a structural and functional paralog with 68% sequence identity to Nck1, and Grb2 (CAG46740.1), which is similar except that it has only two SH3 domains. Even with prolonged exposures there was no evidence for recovery of Nck2 or Grb2 with cytoplasmic CE (Figure 2D). Lastly, the interaction of endogenous Nck1 with endogenous CE was confirmed by a guanylylation assay performed on complexes recovered by immunoprecipitation of cytoplasmic extract from non-transfected cells with anti-Nck1 antibody (Figure 2E).

### Identification of Nck1 in a Complex with Cytoplasmic Capping Enzyme

**Figure 3:**
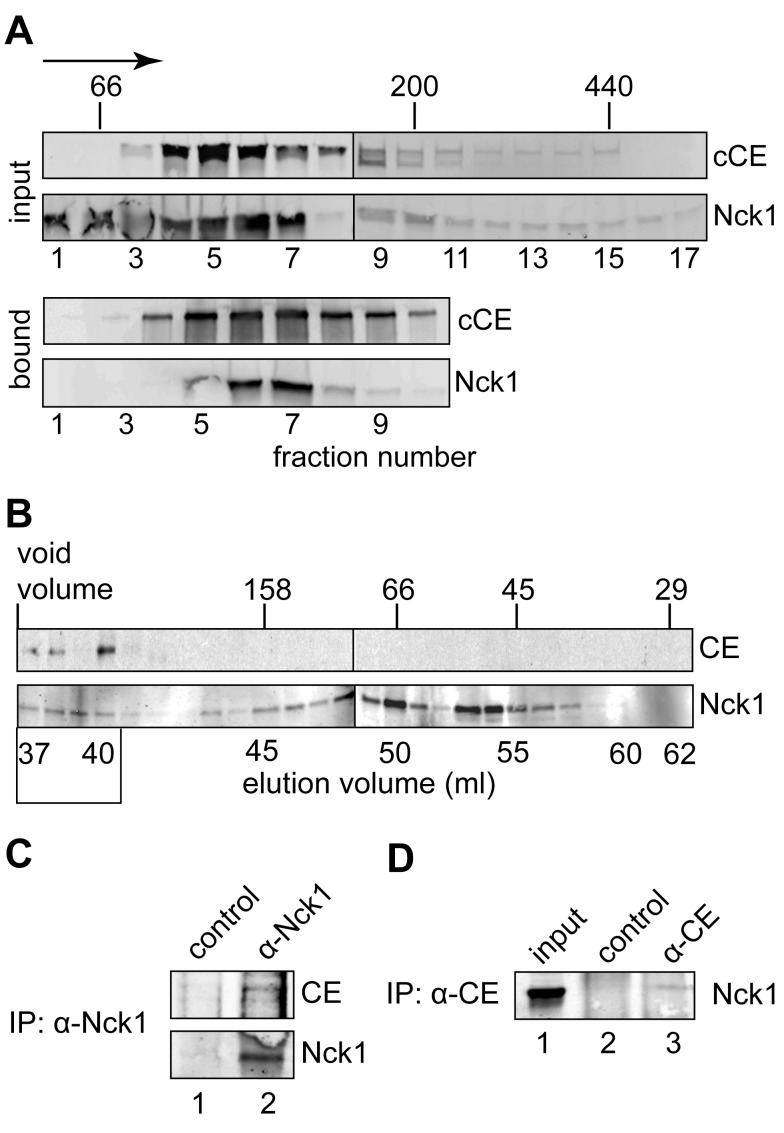
Identification of Nck1 as a component of the cytoplasmic capping complex. (A) Cytoplasmic extract from cells expressing bio-cCE was separated on a 10%–50% glycerol gradient. Fractions containing each of these proteins were identified by Western blotting of input fractions with antibodies to the Myc tag on bio-cCE and to Nck1 (upper 2 panels). Streptavidin beads were used to recover bio-cCE from individual fractions and bound proteins were again analyzed by Western blotting with anti-Myc and anti-Nck1 antibodies (lower two panels). (B) Cytoplasmic extract from non-transfected cells was separated on a calibrated Sephacryl S-200 column. Starting with the void volume individual fractions were collected and analyzed by Western blotting with anti-CE and anti-Nck1 antibodies. The missing CE band in fraction 3 was due to sample loss during loading. (C) The fractions indicated with a box at the bottom of (B) were pooled and immunoprecipitated with anti-Nck1 or control IgG. 20% of the immunoprecipitated sample was used for Western blotting with anti-Nck1 antibody and 70% of the immunoprecipitated sample was used for Western blotting with anti-CE antibody. (D) Cytoplasmic extract from non-transfected cells was immunoprecipitated with anti-CE antibody or control IgG, and the recovered proteins were analyzed by Western blotting with anti-Nck1 antibody.

Previous work showed that CE and the 5′-kinase activity co-sediment on glycerol gradients in a ∼140 kDa cytoplasmic complex [11]. To determine if Nck1 is part of the cytoplasmic capping complex, extract from bio-cCE-expressing cells was separated on a 10%–50% glycerol gradient and Western blotting was used to determine the sedimentation of each of these proteins in the input fractions (Figure 3A, upper panel), and in protein recovered on streptavidin beads (lower panel). A portion of Nck1 overlapped in the input fractions with bio-cCE, and the recovery of Nck1 with bio-cCE on streptavidin beads confirmed its presence in the cytoplasmic capping complex.

We next looked for the evidence of a native complex containing CE bound to Nck1. In the experiment in Figure 3B cytoplasmic extract from non-transfected cells was separated on a calibrated Sephacryl S-200 column, and individual fractions were analyzed by Western blotting for CE and Nck1. Both proteins eluted in the same fractions, at a size estimated from standards to be larger than that seen on glycerol gradients. The difference in size determinations may be due to the shape of the complex or the dissociation of one or more proteins during prolonged sedimentation. Nck1 was also present in later fractions, a result that is consistent with an excess of Nck1 over CE (see Discussion). To determine if CE was bound to Nck1 in the co-eluted fractions these were pooled, immunoprecipitated with control immunoglobulin G (IgG) or anti-Nck1, and the recovered proteins were analyzed by Western blotting with antibodies to both proteins (Figure 3C). The selective recovery of CE with Nck1 confirmed that the native proteins were indeed bound to each other in this complex.

The relative amount of Nck1 bound to CE was estimated by immunoprecipitating cytoplasmic extract from non-transfected cells with anti-CE antibody followed by Western blotting with anti-Nck1 antibody (Figure 3D). On the basis of signal intensity and the amount of protein loaded onto the gel, we estimate that 1% of Nck1 is bound to cytoplasmic CE.

### Nck1 Is the Scaffold for Assembly of the Cytoplasmic Capping Complex

**Figure 4:**
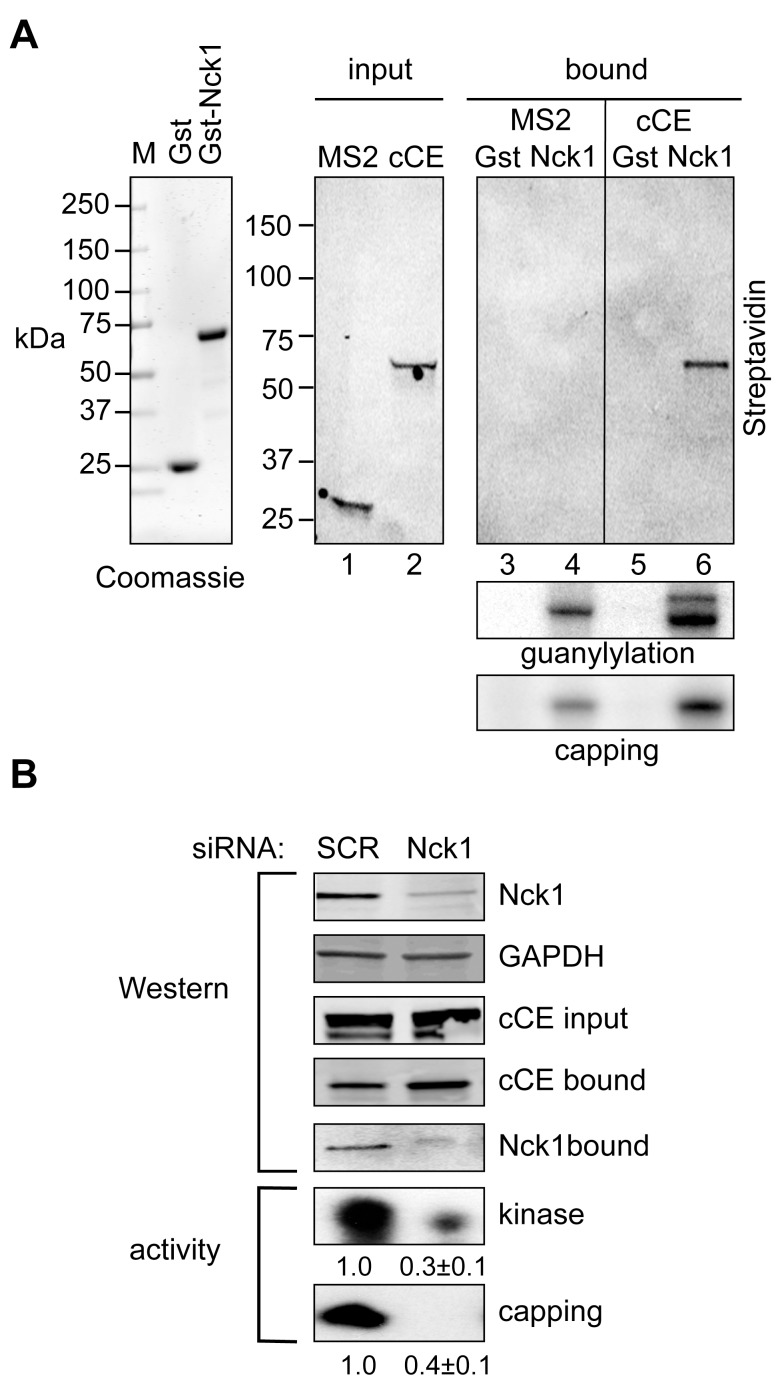
The cytoplasmic capping complex assembles on Nck1. (A) Gst and Gst-Nck1 were expressed in *E. coli* and protein purified on glutathione agarose was analyzed by SDS-PAGE stained with Coomassie Blue (left panel). The middle panel is a Western blot using HRP-streptavidin of cytoplasmic extracts of cells that were transfected with plasmids expressing bio-cCE or MS2-bio. Gst- or Gst-Nck1 bound glutathione beads were added to each of these extracts (right panels) and bound proteins were analyzed by Western blotting with HRP-streptavidin (top panel), for guanylylation activity (middle panel), and for capping activity (bottom panel). The single band in guanylylation assay of MS2-bio expressing cells is endogenous CE, the two bands in bio-cCE expressing cells correspond to endogenous CE and bio-cCE. (B) 293 cells were transfected with Nck1 siRNA or a scrambled control and plasmid expressing bio-cCE. The effectiveness of Nck1 knockdown was determined by Western blotting with anti-Nck1 and anti-GAPDH antibodies, and the expression of bio-cCE and its recovery on streptavidin beads was monitored by Western blotting with anti-Myc antibody. The recovery of Nck1 with bio-cCE was determined by Western blotting with anti-Nck1 antibody (upper panels). In the bottom two panels the recovered complexes were assayed for recovery of 5′-kinase activity and capping activity, normalized to bio-cCE recovery, with the mean ± standard deviation (*n* = 3) shown beneath each autoradiogram. For each of these *p*-value was <0.05 by Student's *t* test.

Because Nck1 lacks catalytic activity its presence in the cytoplasmic capping complex suggested it might act as a scaffold to bring CE together with the 5′-kinase and perhaps other proteins. To test this concept we first asked if a functional complex could assemble *in vitro* on recombinant Nck1. Gst and Gst-Nck1 were expressed in *Escherichia coli* (Figure 4A, left panel) and bound to glutathione beads that were added to pre-cleared extracts from cells expressing bio-cCE or MS2-bio (Figure 4A, middle panel). Selective *in vitro* binding of bio-cCE to Nck1 was demonstrated by Western blotting with HRP-streptavidin (Figure 4A, right panel). Perhaps of greater importance, guanylylation assay showed that CE present in each of the extracts also bound selectively to Nck1 (Figure 4A, right middle panel, lanes 4 and 6). Finally, capping assay was performed on the bead-bound proteins to determine if all of the activities (i.e., CE plus the 5′-kinase) can assemble *in vitro* on Nck1. Bead-bound proteins were incubated with 5′-monophosphate RNA, ATP, and α-[^32^P]GTP, and the products were separated on a denaturing gel. The GMP labeling of 5′-monophosphate RNA by proteins recovered with Gst-Nck1 but not with Gst alone (Figure 4A right, bottom panel, lanes 4 and 6) supports the hypothesis that Nck1 functions as a scaffold for assembly of the cytoplasmic capping complex.

The preceding data also suggest that CE and the 5′-monophosphate kinase each bind to Nck1 but not to one another. If so, Nck1 knockdown should reduce the amount of kinase and capping activity recovered with bio-cCE. In the experiment in Figure 4B cells were transfected with bio-cCE and Nck1 siRNA or a scrambled control, and protein recovered on streptavidin beads was assayed by Western blotting, and for kinase activity and capping activity. Nck1 knockdown had no impact on the amount of bio-cCE or its recovery on streptavidin beads. However, significantly less kinase and capping activity were recovered in cells knocked down for Nck1 compared to the scrambled control (lower panels). Together with results in Figure 4A these data point to Nck1 as the scaffold that brings cytoplasmic CE together with the 5′-kinase to form a functional capping complex.

### CE and the 5′-Kinase Activity Bind to Adjacent SH3 Domains of Nck1

**Figure 5:**
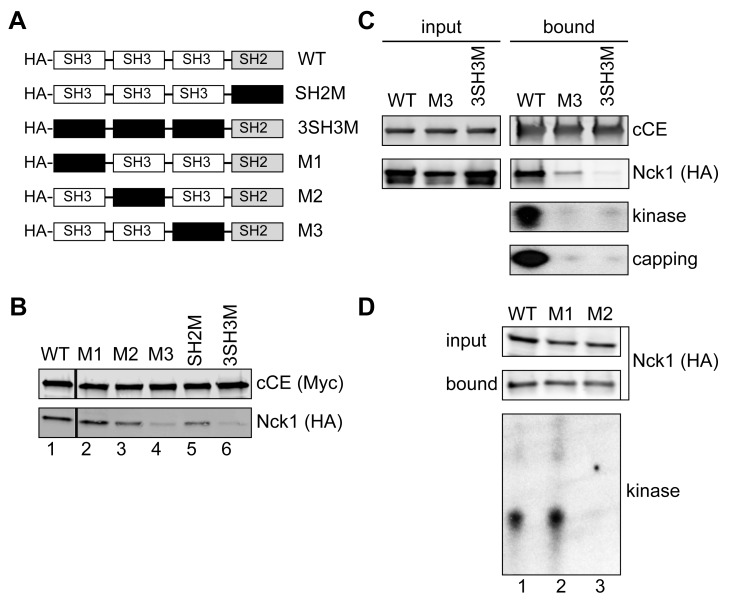
Identification of the CE and 5′-kinase binding domains. (A) The organization of Nck1 (wild type [WT]) is shown together with a series of plasmids expressing HA-tagged forms with inactivating mutations (black box) in each of the functional domains. (B) HEK293 cells were co-transfected with plasmids expressing the indicated forms of Nck1 and bio-cCE. Protein recovered on streptavidin beads was analyzed by Western blotting with antibodies to the Myc tag on bio-cCE and the HA tag on Nck1. (C) Cells were co-transfected with plasmids expressing bio-cCE and HA-tagged wild type Nck1 (WT) or Nck1 with inactivating mutations in the third SH3 domain (M3) or all 3 SH3 domains (3SH3M). Protein recovered on streptavidin beads was analyzed by Western blotting with Alexafluor800-coupled streptavidin (cCE) and anti-HA (Nck1) antibody. Kinase activity was assayed by incubating the recovered proteins with a 23 nt 5′-monophosphate RNA and γ-[^32^P]ATP, and capping activity was assayed by incubating recovered proteins with with ATP and α-[^32^P]GTP. The products of each reaction were separated on a denaturing polyacrylamide/urea gel and visualized by autoradiography. (D) HEK293 cells were transfected with the plasmids expressing HA-tagged forms of wild-type Nck1, or Nck1 with inactivating mutations in the first (M1) and second (M2) SH3 domains. Complexes recovered on anti-HA beads were analyzed by Western blotting (upper panels), and for 5′-kinase activity as in (C).

The CE-binding domain on Nck1 was identified by co-transfecting cells with bio-cCE and a panel of Nck1 constructs with inactivating mutations in each of the functional domains (Figure 5A) [20]. The almost complete loss of Nck1 mutated in the third SH3 domain from protein recovered on streptavidin beads identified this as the CE binding site (Figure 5B, M3, lane 4, 3SH3M, lane 6). The functional impact of this mutation was determined by assaying the recovery of kinase and capping activity with bio-cCE from cells expressing wild-type Nck1 or Nck1 with the CE-binding domain mutation (Figure 5C). In both cases the loss of Nck1 binding was matched by a similar loss in recovery of kinase activity and capping activity, thus confirming that Nck1 acts as a scaffold to bring the 5′-kinase together with cytoplasmic CE to form the cytoplasmic capping complex.

To determine which of the other SH3 domains binds the 5′-kinase activity cells we transfected cells with plasmids expressing HA-tagged wild-type Nck1, or Nck1 mutated in the first SH3 (M1) or second (M2) SH3 domain (Figure 5D, upper panel). Proteins were recovered on anti-HA beads and assayed for kinase activity by incubation with γ-[^32^P]ATP and a 23 nt 5′-monophosphate RNA, followed by denaturing gel electrophoresis of recovered RNA. 5′ kinase activity was recovered with wild-type Nck1 and Nck1 mutated in the first SH3 domain (M1), but not with Nck1 mutated in the second SH3 domain (M2, Figure 5D, lower panel). Thus, the core of the cytoplasmic capping complex consists of CE and the 5′-kinase bound to adjacent sites on Nck1.

### Nck1 Functions in Cap Homeostasis

We next sought to build on our success in knocking down Nck1 (Figure 4B) to demonstrate a functional role for Nck1 in cap homeostasis. However, Nck1 knockdown resulted in a general decrease in the steady-state level of every transcript examined, regardless of classification with respect to cytoplasmic capping (Figure S5). The reason for this is not known, but it does not appear to be related to cell viability, as this was unaffected by Nck1 knockdown (Figure S6). As an alternative we asked whether cap homeostasis could be disrupted by overexpression of Nck1 with inactivating mutations in the CE and 5′-kinase binding domains.

**Figure 6:**
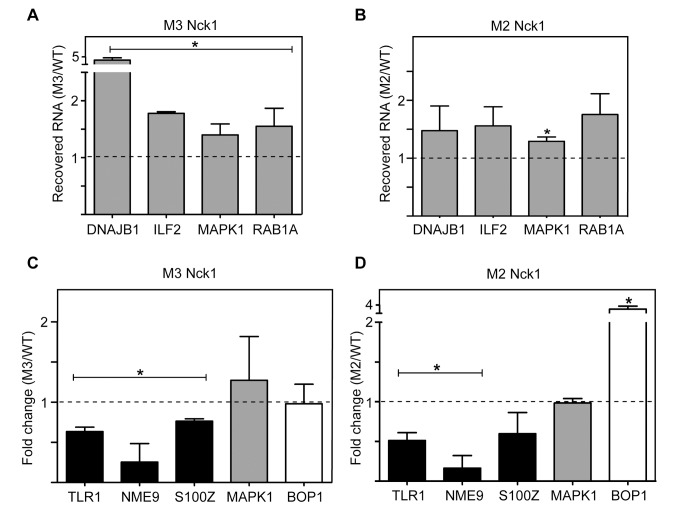
A functional role for Nck1 in cap homeostasis. Triplicate cultures of U2OS cells were transfected with plasmids expressing HA-tagged wild-type Nck1, Nck1 mutated in the CE-binding domain (M3), or the 5′-kinase-binding domain (M2). Western blots showing overexpression of each of these proteins are in Figure S4. The appearance of uncapped forms of transcripts in the “capping inhibited” pool (grey bars) was determined by their recovery on streptavidin beads after ligation of an RNA adapter and hybridization to a biotinylated antisense DNA oligonucleotide [13]. Each preparation contained an equal amount of uncapped human β-globin mRNA as an internal control and RNA recovered from M3 (A) and M2 (B) expressing cells was analyzed by qRT-PCR for four transcripts that accumulate uncapped forms in cells that are inhibited for cytoplasmic capping (DNAJB1, ILF2, MAPK1, RAB1A). The results are normalized to the signal from cells expressing wild-type Nck1. RNA from M3 (C) or M2 (D) expressing cells was also analyzed by qRT-PCR for three transcripts of the “uninduced” pool whose steady state levels are reduced when cytoplasmic capping is inhibited (TLR1, NME9, S100Z, black bars), one of the transcripts examined in a and b (MAPK1, grey bars), and a control transcript (BOP1, white bars). The results are presented as fold change with respect to wild-type Nck1 and are presented as mean ± standard deviation. The asterisk (*) indicates *p*<0.05 by Student's unpaired two-tailed *t* test.

As noted in the Introduction, cytoplasmic capping targets can be categorized by differences in their behavior when cytoplasmic capping is inhibited. The “capping inhibited” pool make up the most obvious targets because stable uncapped forms of these transcripts appear when cytoplasmic capping is inhibited. Triplicate cultures of U2OS cells were transfected with plasmids expressing wild-type Nck1, or the M2 and M3 forms of Nck1, and their overexpression with respect to endogenous Nck1 was confirmed by Western blotting (Figure S7). We also confirmed that their overexpression did not have an inhibitory impact on their steady-state levels as seen with Nck1 knockdown. The appearance of uncapped transcripts was determined using an assay from our previous study in which these are ligated to an RNA adapter, hybridized to a biotinylated antisense DNA oligonucleotide, and recovered on streptavidin beads [21]. Each preparation included an internal control of uncapped β-globin RNA. In agreement with a central role for Nck1 in cap homeostasis, overexpression of Nck1 mutated in the CE (Figure 6A, M3) or 5′-kinase binding domain (Figure 6B, M2) resulted in the appearance of uncapped forms of each of four “capping inhibited” targets (DNAJB1, NM_006145; ILF2, NM_004515.3; MAPK1, NM_002745.4; and RAB1A, NM_004161.4).

Inhibition of cytoplasmic capping also results in the Xrn1-mediated degradation of natively uncapped transcripts in the “uninduced” pool. The impact of overexpressing the M3 (Figure 6C) and the M2 (Figure 6D) forms of Nck1 was examined for three of these transcripts (TLR1, NM_003263.3; NME9, NM_178130.2; S100Z, NM_130772.3). The steady-state level of each target RNA was reduced compared to wild-type control, again confirming cap homeostasis is inhibited by overexpression of Nck1 with mutations in the CE or 5′-kinase binding domain. The mutant forms of Nck1 had little impact on the steady-state level of MAPK1, a result that is consistent with the stability of the uncapped forms of this class of transcripts. We also looked at the impact of each of these proteins on steady-state levels of BOP1 (NM_015201.3), one of the control transcripts whose cap status is unaffected by changes in cytoplasmic capping. M3 overexpression had no impact on the level of BOP1 mRNA; however, BOP1 was unexpectedly increased in cells that overexpress the M2 form of Nck1. The reason for this is not known, but we suspect it may be a consequence of interfering with one of the other pathways in which Nck1 participates.

## Discussion

Prior to this study the functional domains of mammalian capping enzyme were limited to the N-terminal triphosphatase domain and the C-terminal guanylyltransferase domain. Our results add a third domain at the C-terminus of the mammalian protein whose binding to the third SH3 domain of Nck1 functions in the assembly of the cytoplasmic capping complex. In the course of this work we also discovered that C-terminal extensions (such as the FLAG tag on CEΔNLS+NES, Figure 1C and K294A) interfere with CE binding to Nck1. Because CE and the 5′-kinase activity bind to Nck1 rather than to each other this may explain why the cytoplasmic capping targets identified in Mukherjee and colleagues [13] have 5′-monophosphate ends rather than 5′-diphosphate ends as might be expected if these proteins interacted directly.

We realized early on that knowing the targets of cytoplasmic capping was a necessary first step toward validating the identity of proteins in the cytoplasmic capping complex. Results in [13] grouped the targets of cytoplasmic capping into three broad classes on the basis of the relative stability of their uncapped forms. Overexpression of Nck1 with inactivating mutations in the CE (Figure 6A) or the 5′-kinase-binding domains (Figure 6B) resulted in the appearance of uncapped forms of transcripts that also appear when cytoplasmic capping was blocked by induction of the inactive K294A form of cytoplasmic CE. Other cytoplasmic capping targets have natively uncapped forms that are degraded when cytoplasmic capping is inhibited. Together with the appearance of stable uncapped forms of the “capping inhibited” transcripts, the lower steady-state levels of these RNAs in cells expressing Nck1 with inactivating mutations in the CE (Figure 6C) or 5′-kinase (Figure 6D) binding sites provided *in vivo* confirmation of an essential role for Nck1 in cytoplasmic capping. Together, they confirm that Nck1 is essential for the assembly of the cytoplasmic capping complex and for cap homeostasis.

**Figure 7:**
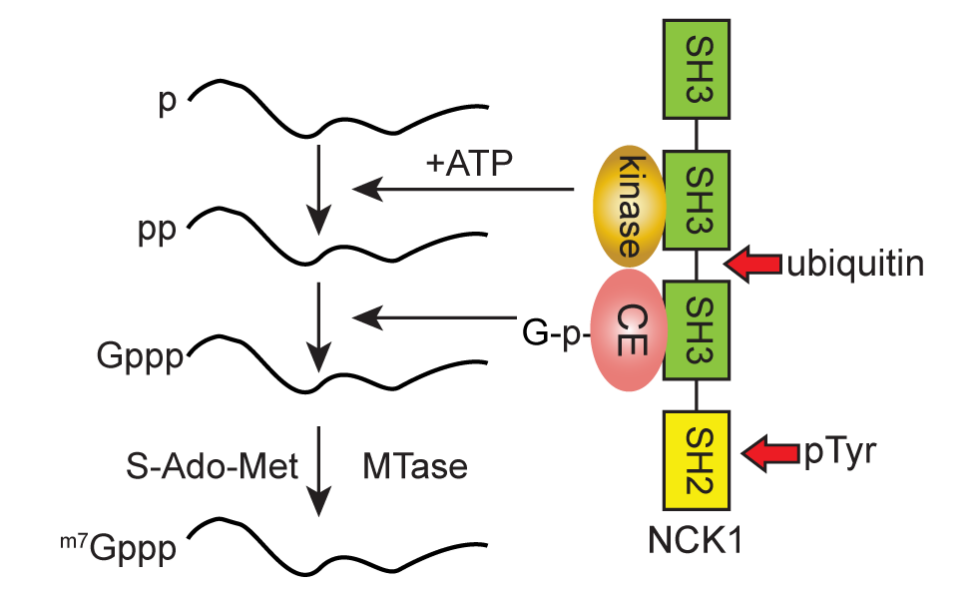
Model for assembly of the cytoplasmic capping complex. In this model Nck1 serves as the scaffold for assembly of the cytoplasmic capping complex, with the 5′-kinase and CE juxtaposed by binding to adjacent domains. The ubiquitination site between the second and third SH3 and the SH2 domain for binding tyrosine phosphoproteins (pTyr) are indicated by red arrows. It has yet to be determined how the methyltransferase joins the complex to complete the reaction.

Nck1 is a ubiquitously expressed cytoplasmic protein that is best known for its role in transducing tyrosine kinase signaling [22],[23]. It also functions in the resolution of endoplasmic reticulum stress [24],[25] and it stimulates translation by binding to eIF2β [17]. On the organismal level Nck1 is required for proper mesoderm development [18] and in establishing neuronal circuitry [26]. Nck1 and its paralog Nck2 are elevated in many cancers, and their overexpression promotes malignant transformation [27]. Figure 7 presents a model of our current understanding of the cytoplasmic capping complex. By binding to adjacent SH3 domains, Nck1 juxtaposes CE and the 5′-kinase activity in a manner that is likely to facilitate the generation of a diphosphate capping substrate and the transfer of GMP onto this. Although Nck1 and Nck2 share 68% sequence identity CE only binds to Nck1 (Figure 2C). We know from results in [11] that the products of cytoplasmic capping are properly methylated, but have yet to confirm the identities of the 5′-kinase activity or the cap methyltransferase.

Approximately 25% of the 5′ ends identified by CAGE analysis of mammalian transcriptomes map within downstream exons rather than transcription start sites [7],[28]. Those findings are consistent with the recent identification of unique protein products translated from small ORFs located downstream of canonical start sites [29]–[32]. Our findings suggest that the absence of downstream CAGE tags in the *Drosophila* transcriptome [6] results from the inability of *Drosophila* CE to bind Nck1 and participate in the cytoplasmic capping complex. The presence of Nck1 at the core of the cytoplasmic capping complex also suggests that cytoplasmic capping, and perhaps the proteins translated from recapped transcripts, may vary in response to different stimuli, for example by activation of a particular receptor tyrosine kinase. Adding to this complexity, the amount of Nck1 is also regulated by the ubiquitin/proteasome pathway [33]. Ubquitination by the c-Cbl E3 ubiquitin ligase targets Nck1 for degradation by the proteasome, which in turn may impact cytoplasmic capping by reducing the amount of Nck1 that is available for bringing cytoplasmic CE together with the 5′-kinase. Nck1 ubiquitination is inhibited by the binding of synaptopodin, a proline-rich actin binding protein, to the same site on Nck1 as the 5′-kinase activity. These findings raise the possibility of competition by these proteins, and of a link between cytoplasmic capping and the cytoskeleton.

## Materials and Methods

### Plasmid Constructs

pcDNA3 myc-mCE [11] was used as a template to amplify myc-mCEΔ25C using the primers T7 and YO125 containing Kpn1 and Apa1 sites, respectively. The amplified PCR product was digested with Kpn1 and Apa1 and then ligated into similarly digested pcDNA3. The C-terminal FLAG tag was removed from pcDNA4/TO-myc-NES-mCEΔNLS-Flag [11] by amplifying the region containing Myc-cCE with primers Tevbio-cCE-F and Tevbio-cCE-R. This was further modified by addition of a C-terminal tag containing a site for cleavage by Tev protease and a peptide that is biotinylated *in vivo* [34]. The plasmid pFA6a-HTB-hphMX4 containing this sequence was provided by Peter Kaiser (University of California, Irvine). The biotinylation tag sequence was amplified from this plasmid using cCE-TevBio-F and cCE-TevBio-R. The PCR products from the preceding two reactions were mixed together and PCR amplified with primers cCE-F and R-Tev Bio to create Myc-cCE-Tev-Biotin. This was digested by Kpn I and Apa I and cloned in similarly digested pcDNA3/TO vector yielding pcDNA3/TO myc-cCE-bio (cCE-bio). To generate CE with an N-terminal biotinylation tag (bio-cCE), the sequence corresponding to the tag only (without the Tev cleavage site) was amplified from the above template with primers Bio RP and Bio FP. This tag was introduced into pcDNA3 myc-NES-mCEΔNLS, pcDNA3 myc-NES-mCE (K294A) ΔNLS [11] and pcDNA3 myc-mCEΔ25C using the In-Fusion HD cloning kit (Clontech). The NES from construct used in our previous work was added to pcDNA3 bio-myc-mCEΔ25C to generate pcDNA3 bio-myc-NES-mCEΔ25C (bio-cCEΔ25C). Bio-cCEΔpro was generated from bio-cCE by site directed mutagenesis using the QuikChange Site-Directed Mutagenesis kit (Stratagene) with oligos Amp-R (Stratagene) and cCE-ΔPPP. The plasmid for expression of GFP (pcDNA4/TO myc-GFP-Flag) was described previously [11]. All constructs were verified by sequencing. Sequences of oligos used in the study are listed in Table S1. The human Nck1 constructs were described in [20] and kindly provided by Wei Li and Louise Larose [17]. In these the SH2 domain in SH2M was inactivated by an arginine-to-lysine mutation in the sequence FLVRES, and the SH3 domains in M1, M2, M3, and 3SH3M were each inactivated by changing the first tryptophan in the WW motif to lysine. The MS2-Biotin construct was provided by Marion Waterman, University of California, Irvine [19].

### Cell Culture and Transient Transfection

U2OS and HEK-293 cells were grown in McCoy's 5A medium (Invitrogen) containing 10% fetal bovine serum. 1×10^6^ HEK293 or 2×10^6^ U2OS cells in log phase growth were transfected with 8 µg (total) of plasmid DNA using FuGENE 6 (Promega) following the manufacturer's protocol. Cells were harvested 36 h post-transfection. Transfection efficiency (typically 95% for 293 cells and 70% for U2OS cells) was determined by parallel transfection with a GFP-expressing plasmid.

### Preparation of Cytoplasmic Extract and Isolation of Cytoplasmic RNA

Cells were lysed using 1× lysis buffer (20 mM Tris-HCl [pH 7.5], 10 mM NaCl, 10 mM MgCl_2_, 10 mM KCl, 0.2% NP40, 1 mM PMSF [Sigma], 1× protease inhibitor [Sigma], 1× phosphatase inhibitor cocktail II and III [Sigma], and 80 units/ml RNase out [Invitrogen]). These were placed on ice for 5 min, the tubes were gently flicked and incubated on ice for an additional 5 min. The lysates were centrifuged at 5,000 *g* at 4°C for 10 min to pellet the nuclei, and the supernatant (cytoplasmic) fractions were transferred to chilled microcentrifuge tubes. Cell lysate was used directly for Immunoprecipitation and Gst pull-down assays except for experiments where extracts were treated with micrococcal nuclease to remove nucleic acid. RNA was recovered from cytoplasmic fractions with Trizol reagent (Invitrogen) as per the manufacturer's instructions. The recovered RNA was treated with DNase I and poly(A) RNA was selected using Dynabeads mRNA DIRECT kit (Invitrogen) according to the manufacturer's instructions. Cells were fractionated into cytoplasmic and nuclear fractions by NE-PER kit using manufacturer's (Pierce) protocol for gel filtration and subcellular localization of Nck1.

### Glycerol Gradients

Cytoplasmic extracts from transiently transfected HEK293 were layered onto freshly prepared 10%–50% linear glycerol gradients and centrifuged at 200,000 *g* for 22 h at 4°C. Each experiment included a gradient containing gel filtration standards as described in [11] for use as reference points for determining size as a function of position within the gradient. 500 µl fractions were collected from the top of the gradient and stored at −80°C. Protein present in 100 µl of each fraction was first recovered by precipitation with ten volumes of ethanol prior to Western blot analysis, and remaining fractions were used to recover cytoplasmic capping enzyme on streptavidin beads.

### Gel Filtration

Two mg of HEK293 cell cytoplasmic extract was filtered through a 0.2 µ low protein binding filter (Millipore) and then loaded at 4°C into a calibrated HiPrep 16/60 Sephacryl S-200 High Resolution column (GE) in 15 mM Tris-HCl (pH 7.5), 150 mM NaCl. The column was developed in the same buffer at a flow rate of 0.5 ml/min, and starting from the void volume (elution volume 37 ml) 0.5 ml fractions were collected up to an elution volume of 62 ml in order to separate monomeric proteins as small as 29 kDa. 250 µl from each fraction was TCA precipitated and analyzed by Western blotting with anti-CE and anti-Nck1 antibodies. The remaining 250 µl of the four fractions indicated with a box in Figure 3B were pooled and immunoprecipitated with control IgG or anti-Nck1 antibody and Dynabeads protein G. The recovered proteins were analyzed by Western blotting with anti-CE and anti-Nck1 antibodies.

### Expression and Purification of Recombinant GST-Nck1 and GST Pull Down

*E.coli* BL21(DE3)pLysS cells (Promega) were transformed with plasmid pGEX-2TK-Nck1or pGEX-2TK. These were grown in Luria Bertani broth containing 100 µg/ml ampicillin and 50 µg/ml chloramphenicol. Cells were induced at an OD_600_ of 0.5 with 0.5 mM IPTG at 18°C and cultured overnight. Cells were lysed in GST-lysis buffer (20 mM Tris-HCl [pH 7.5], 20 mM NaCl, 1 mM DTT, 1 mM PMSF, 1% Triton-X). Gst or Gst-Nck1 expressed in *E. coli* were bound with gentle rocking to glutathione Sepharose for 2 h at 4°C. The beads were washed 5× with Gst wash buffer (25 mM Tris-HCl [pH 7.5], 150 mM NaCl, 0.1% NP40) to remove unbound proteins and stored at 4°C in the same buffer containing 20% glycerol and 1 mM phenylmethylsulfonyl fluoride. Cytoplasmic extracts were prepared 36 h after transfection of HEK293 with plasmids expressing bio-cCE or MS2-bio. Nonspecific proteins were removed by incubating each extract with glutathione-Sepharose, and 5 mg of pre-cleared extract was incubated with rocking for 2 h at 4°C with Gst- or Gst-Nck1-bound beads. These were recovered by centrifugation and unbound proteins were removed by five washes with Gst wash buffer. The bead-bound proteins were analyzed by Western blotting, and for guanylylation and capping activity [11].

### Immunoprecipitation, Streptavidin Recovery, *In Vitro* Guanylation, Kinase, and Capping Assays

For immunoprecipitation reactions, cytoplasmic extract was pre-cleared with mouse IgG antibody coupled beads (Cell Signaling). The pre-cleared lysate was incubated with anti-Myc or HA monoclonal antibody coupled beads for 2 h at 4°C in a rocking platform. Biotinylated proteins were recovered by incubating extracts for the same time as above with Streptavidin Dynabeads (T1, Invitrogen). For immunoprecipitation with anti-Nck1 or anti-CE antibodies cytoplasmic extract was first pre-cleared with Protein G Dynabeads (Invitrogen), followed by addition of control rabbit IgG or antibody IgG bound Protein G Dynabeads. The reactions were incubated overnight at 4°C in a rocking platform before washing and recovery of antibody-bound complexes. In each experiment bead bound proteins were washed and processed for western blotting or *in vitro* guanylylation, kinase and capping reactions [11].

### Antibodies

Mouse anti-Myc monoclonal antibody, rabbit IgG, anti-Myc coupled agarose beads and rabbit anti-HA antibodies were obtained from Santa Cruz Biotechnology. Anti-HA antibody coupled magnetic beads were purchased from Pierce. Mouse monoclonal antibodies to β-tubulin and GAPDH were obtained from Sigma. Mouse anti-HA monoclonal antibody was purchased from Roche. Monoclonal Rabbit anti-Nck1 was obtained from Cell Signaling and polyclonal rabbit anti-Nck1 antibody used for immunoprecipitation was obtained from Millipore. Rabbit polyclonal antibody to Nck2 was obtained from Upstate Biotechnology and rabbit polyclonal antibody to CE was purchased from Novus. Rabbit polyclonal antibody to Grb2 was provided by Ramesh K Ganju, Ohio State University. Conformation specific mouse anti-rabbit antibody was obtained from Cell Signaling. Alexafluor coupled goat anti-rabbit IgG (680), goat anti-mouse (800), goat anti-rabbit IgG (488), goat anti-mouse IgG (594) and streptavidin (800) were purchased from Molecular Probes (Invitrogen). HRP coupled goat anti- rabbit antibody and HRP-streptavidin were obtained from Santa Cruz Biotechnology.

### Western Blot Analysis

5% input (otherwise indicated) and 70% of each immunoprecipitated sample were denatured in 2× Laemmli buffer (Bio-Rad Laboratories) containing β-mercaptoethanol and incubated at 95°C for 5 min prior to electrophoresis on 10% Mini-PROTEAN TGX precast gels (Bio-Rad Laboratories). Proteins were transferred onto Immobilon-P PVDF membrane (EMD Millipore), which were blocked using 3% bovine serum albumin in Tris-buffered saline (TBS). This was followed by incubation with primary antibody for 2 h in blocking solution containing 0.05% Tween-20 (TBS-T). These were then washed with TBS-T, incubated for 1 h with HRP or Alexafluor-coupled secondary antibody (1∶10,000 dilutions), and visualized on X-ray film (GeneMate) after detection with ECL-plus detection system (GE Healthcare), or with a Licor Odyssey imager. To prevent detection of antibody denatured chains in immunoblots, conformation specific mouse anti-rabbit antibody was used following incubation with primary antibody. The antibody dilutions used for Western blots are as follows: NCK1, 1∶2,000; NCK2, 1∶2,000; Grb2, 1∶1,000; HA, 1∶2,000; Myc, 1∶1,000; GAPDH, 1∶5,000; β-tubulin, 1∶5,000; Streptavidin HRP, 1∶10,000; conformation specific mouse anti-rabbit antibody, 1∶2,000.

### Immunofluorescence

U2OS cells grown on coverslips were fixed with methanol at −20°C for 10 min and probed with a 1∶50 dilution of rabbit anti-Nck1 and a 1∶200 dilution of mouse anti-α tubulin. Secondary antibodies consisting of Alexafluor 488 goat anti-rabbit IgG or Alexafluor 594 anti-mouse IgG were used at 1∶1,000 dilution. All images were acquired at ambient temperature using an Olympus IX-81 microscope, with 100× Plan Apo oil immersion objective (1.4 numerical aperture) and a QCAM Retiga Exi FAST 1394 camera, and analyzed using the Slidebook software package (Intelligent Imaging Innovations).

### Quantitative Reverse Transcriptase-PCR and Physical Recovery of Uncapped RNA

A total of 50 ng of cytoplasmic poly (A) RNA was used to synthesize cDNA with Superscript III reverse transcriptase (Invitrogen) according to the manufacturer's instructions. q-PCR was performed with 2×Sensi-FAST Sybr No Rox Mix (Bioline) using an Illumina Eco system. Uncapped RNAs were recovered by a ligation based approach as described in [21] and analyzed by qRT-PCR as described in [13].

### Nck1 Knockdown

HEK293 or U2OS cells were transfected with 10 nM of Nck1 siRNA (J006354-09, Dharmacon) and a scramble control (Dharmacon) using Lipofectamine RNAimax (Invitrogen) according to the manufacturer's protocol. Cells were harvested 72 h after transfection. Knockdown efficiency was monitored by Western blotting with rabbit anti-Nck1 antibody. The impact of Nck1 knockdown on transcript levels was determined by qRT-PCR using primers listed in [13]. The impact of Nck1 knockdown on assembly of the cytoplasmic capping complex was determined by recovery with bio-cCE that was introduced into cells 48 h after transfection with Nck1 siRNA and 12 h before recovering complexes on streptavidin beads.

### Statistical Analysis

Data are shown as the representative result or as mean of at least three independent experiments ± standard deviation. Statistical analyses were performed using Student's unpaired two-tailed *t* test. Differences were considered significant at *p*<0.05. Graphs were generated using GraphPad Prism 5 (GraphPad Software, Inc.) and bars represent standard deviation.

## Supporting Information

Figure S1 **Alignment of human, mouse, and** ***Drosophila*** **capping enzyme.** The capping enzyme sequences from the indicated species were aligned using CLUSTALW. The proline-rich sequences of human and mouse are highlighted in yellow.
(TIF)

Figure S2 **Impact of C-terminal CE modifications on the recovery of kinase activity.** The different forms of CE shown in (A) were recovered as described in the legend to Figure 1. (B) CE recovery was monitored by guanylylation activity. (C) The recovered proteins were assayed for kinase activity by incubating with a 23 nt 5′-monophosphate RNA and γ-[^32^P]ATP. To control for the presence of contaminating RNase activity a [^32^P]labeled capped human β-globin transcript was added to each of the reactions. Its recovery is indicated at the top of the gel. The impact of C-terminal modifications on recovered kinase activity were compared and quantified as in Figure 1C. For each of the comparisons the *p*-value was <0.05 by Student's *t* test.
(TIF)

Figure S3 **Nck1 is a cytoplasmic protein.** (A) 50 µg of each of nuclear and cytoplasmic extract was analyzed by Western blotting with a monoclonal rabbit anti-Nck1 antibody (upper panel) and a polyclonal rabbit anti-U2AF65 antibody. (B) U2OS cells were stained with rabbit anti-Nck1 monoclonal antibody and mouse anti-tubulin monoclonal antibody. Nck1 and tubulin were visualized with Alexafluor 488- or 594-coupled goat anti-rabbit and goat anti-mouse antibodies, respectively. The white bar indicates 5 µm.
(TIF)

Figure S4 **The active site K294A mutation does not affect binding of CE to Nck1.** HEK293 cells were transfected with the plasmids expressing HA-tagged Nck1 and the proteins shown at the top of the figure. Bio-cCE is wild-type CE with an N-terminal biotinylation tag followed by a Myc tag and bio-K294A is the same protein with a lysine to alanine mutation at the GMP binding site. Expression of each of these proteins is restricted to the cytoplasm by addition of the 15 amino acid HIV Rev nuclear export sequence (black rectangle) and deletion of the four amino acid nuclear export sequence (X). The biotinylated tag is indicated by the blue box. In the lower panels cytoplasmic extracts from these cells were recovered on streptavidin beads and analyzed for biotinylated proteins by Western blotting with Alexafluor 800 coupled streptavidin, and for Nck1 with anti-HA antibody. The GMP-binding activity of recovered protein (guanylylation) was assayed by incubation with α-[^32^P]GTP.
(TIF)

Figure S5 **Impact of Nck1 knockdown on steady-state levels of select transcripts.** Triplicate cultures of U2OS cells were transfected with Nck1 siRNA or a scrambled control (Scr). The effectiveness of the knockdown is shown by Western blot in the upper panel. The indicated transcripts were quantified by qRT-PCR and the data are shown as the relative amount present in Nck1 knockdown cells normalized to that of the scrambled control. The data represent the mean ± standard deviation. **p<0.005 by unpaired two-tailed Student's *t* test.
(TIF)

Figure S6 **Impact of Nck1 knockdown on cell viability.** Triplicate cultures of U2OS cells were transfected with Nck1 siRNA or a scrambled control (Scr). The effectiveness of the knockdown is shown by Western blot in the upper panel. Viability was determined after 72 h by CellTiterGlo assay performed using 1×10^5^ or 1×10^6^ cells. Cells knocked down for Nck1 showed no statistically significant difference in cell viability as determined by two-tailed Student's *t* test.
(TIF)

Figure S7 **Impact of overexpressing Nck1 mutated in the second and third SH3 domains on steady-state levels of capping inhibited target mRNAs.** (A) Triplicate cultures of U2OS cells were transfected with plasmids expressing HA-tagged forms of wild-type Nck1 (WT) or Nck1 with an inactivating mutation in the second SH3 domain (M2) or third SH3 domain (M3). Cytoplasmic extracts from each culture were analyzed by Western blotting with anti-Nck1 antibody (upper panel) or anti-GAPDH (lower panel). (B) The impact of M3 overexpression on DNAJB1, ILF2, MAPK1, and RAB1 mRNA was determined by qRT-PCR performed on cytoplasmic RNA recovered from each of the transfectants in (A). The data are plotted as in Figure 6, with results from M3-expressing cells normalized to results from cells expressing wild-type Nck1. (C) The same analysis of DNAJB1, ILF2, MAPK1, AND RAB1 mRNA was performed on RNA from M2-expressing cells. There was no statistically significant difference between each of the treatments as determined by two-tailed Student's *t* test.
(TIF)

Table S1 **Oligonucleotides and primers used in this study.**
(DOC)

## Abbreviations

CAGE: capped analysis of gene expression
CE: capping enzyme (also RNGTT, RNA guanylyltransferase and 5′-phosphatase)
GMP: guanylic acid
IgG: immunoglobulin G
qRT-PCR: quantitative reverse transcriptase PCR
