# Supplementary figures and images for: The Cytoplasmic Capping Complex Assembles on Adapter Protein Nck1 Bound to the Proline-Rich C-Terminus of Mammalian Capping Enzyme
Source: PLoS Biol. 2014 Aug 19;12(8):e1001933. doi: 10.1371/journal.pbio.1001933 (PMC4138027; doi:10.1371/journal.pbio.1001933)

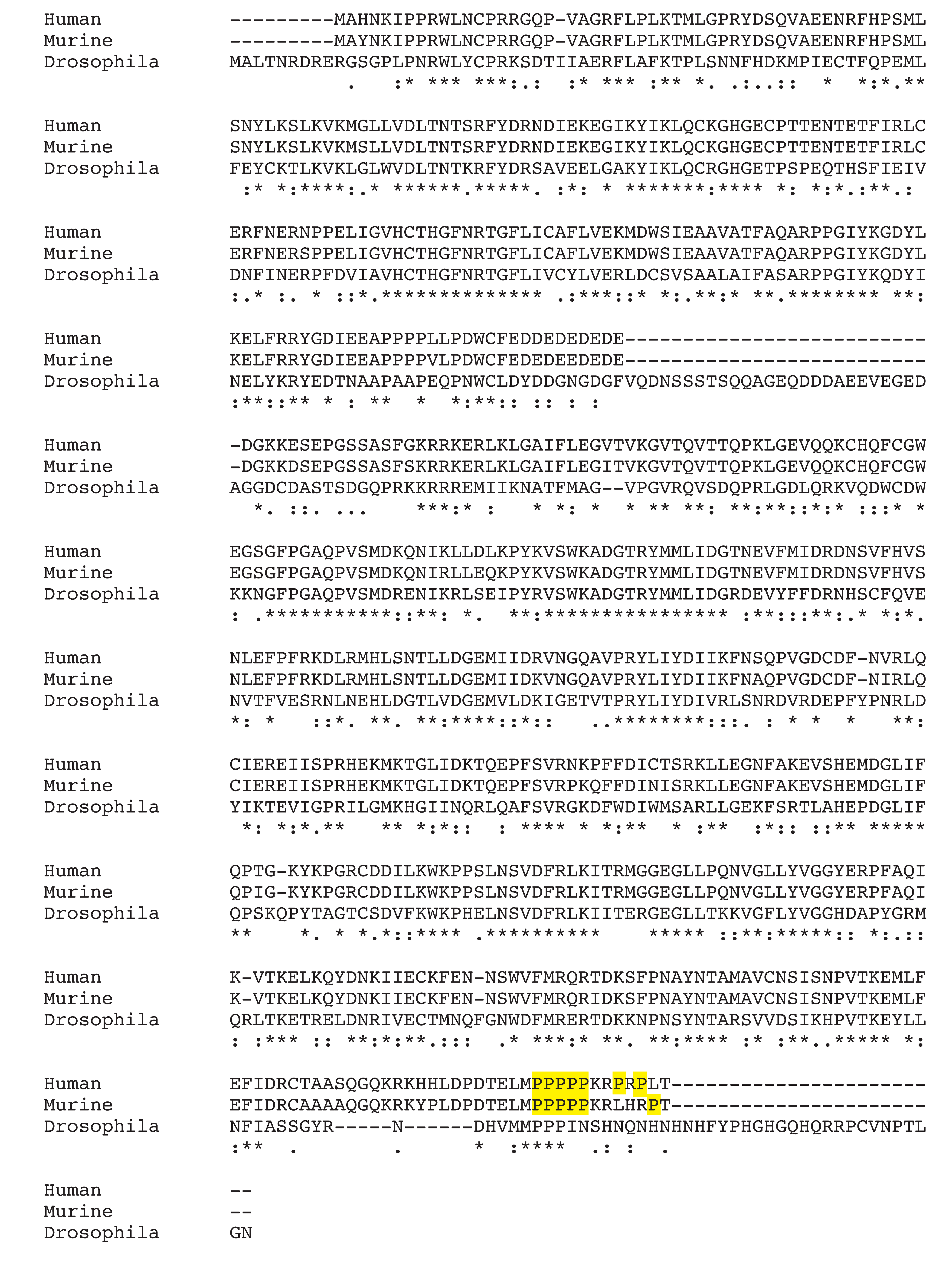

Supplement: Figure S1 — Alignment of human, mouse, and Drosophila capping enzyme. The capping enzyme sequences from the indicated species were aligned using CLUSTALW. The proline-rich sequences of human and mouse are highlighted in yellow. (TIF) [file pbio.1001933.s001.tif]

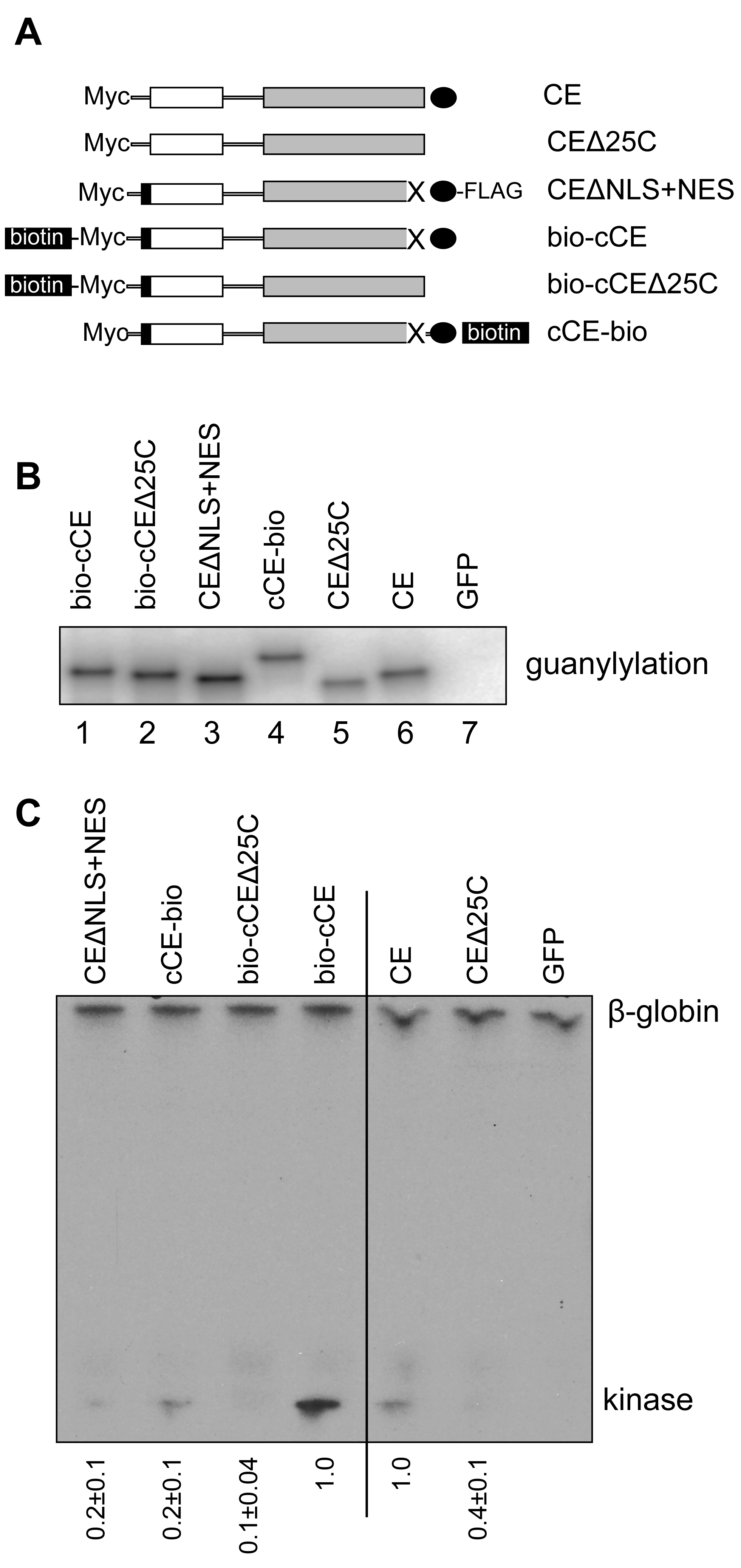

Supplement: Figure S2 — Impact of C-terminal CE modifications on the recovery of kinase activity. The different forms of CE shown in (A) were recovered as described in the legend to Figure 1. (B) CE recovery was monitored by guanylylation activity. (C) The recovered proteins were assayed for kinase activity by incubating with a 23 nt 5′-monophosphate RNA and γ-[32P]ATP. To control for the presence of contaminating RNase activity a [32P]labeled capped human β-globin transcript was added to each of the reactions. Its recovery is indicated at the top of the gel. The impact of C-terminal modifications on recovered kinase activity were compared and quantified as in Figure 1C. For each of the comparisons the p-value was <0.05 by Student's t test. (TIF) [file pbio.1001933.s002.tif]

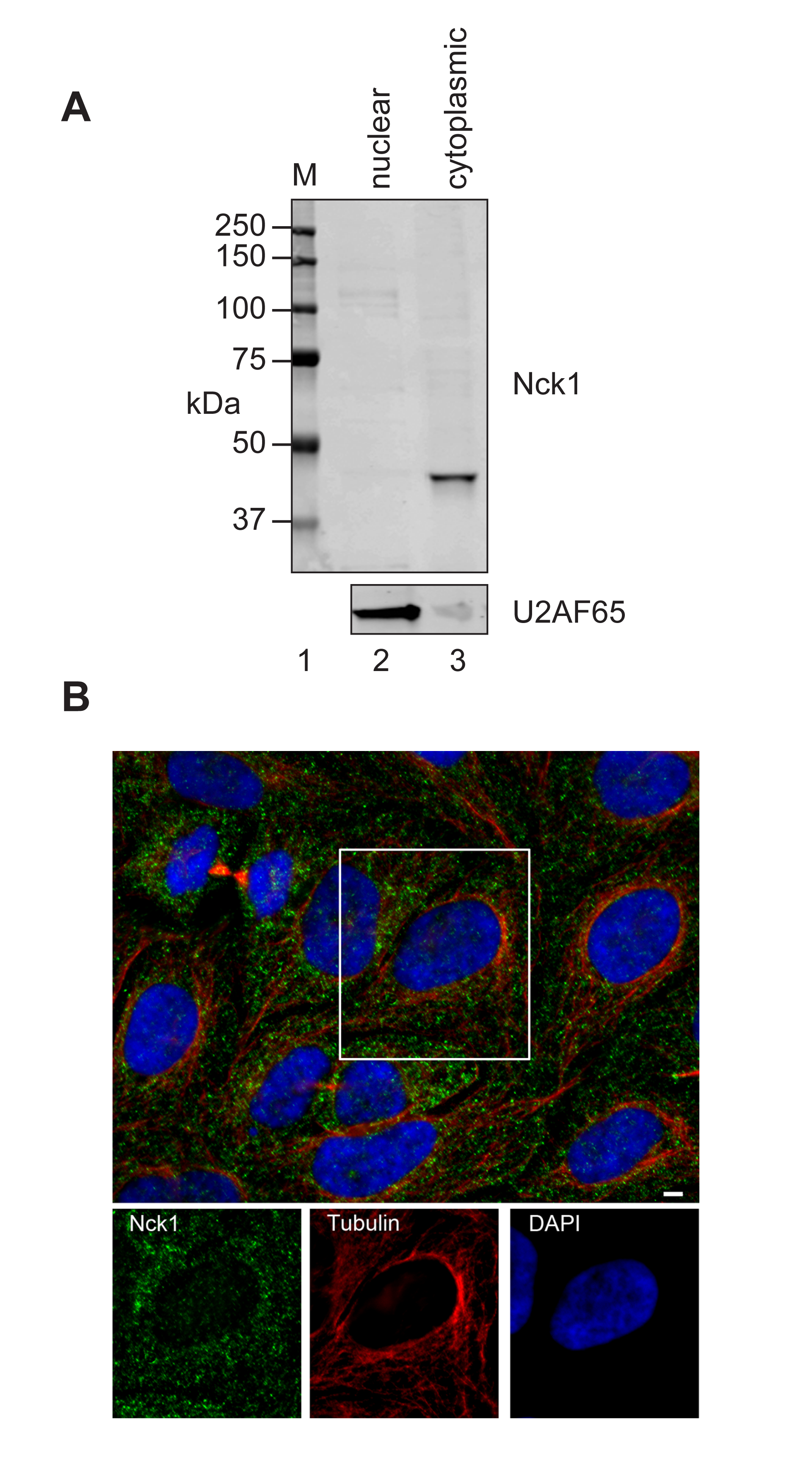

Supplement: Figure S3 — Nck1 is a cytoplasmic protein. (A) 50 µg of each of nuclear and cytoplasmic extract was analyzed by Western blotting with a monoclonal rabbit anti-Nck1 antibody (upper panel) and a polyclonal rabbit anti-U2AF65 antibody. (B) U2OS cells were stained with rabbit anti-Nck1 monoclonal antibody and mouse anti-tubulin monoclonal antibody. Nck1 and tubulin were visualized with Alexafluor 488- or 594-coupled goat anti-rabbit and goat anti-mouse antibodies, respectively. The white bar indicates 5 µm. (TIF) [file pbio.1001933.s003.tif]

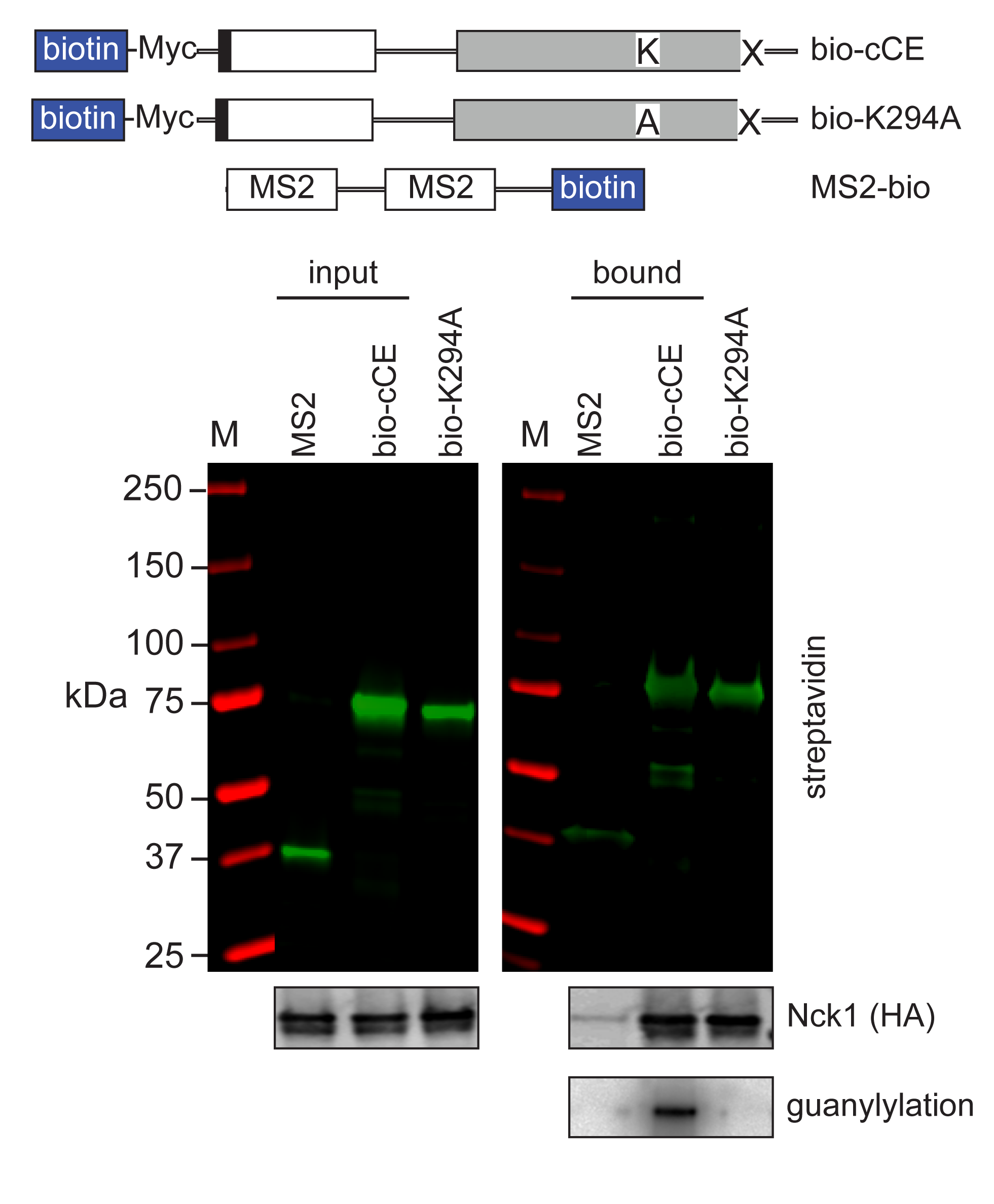

Supplement: Figure S4 — The active site K294A mutation does not affect binding of CE to Nck1. HEK293 cells were transfected with the plasmids expressing HA-tagged Nck1 and the proteins shown at the top of the figure. Bio-cCE is wild-type CE with an N-terminal biotinylation tag followed by a Myc tag and bio-K294A is the same protein with a lysine to alanine mutation at the GMP binding site. Expression of each of these proteins is restricted to the cytoplasm by addition of the 15 amino acid HIV Rev nuclear export sequence (black rectangle) and deletion of the four amino acid nuclear export sequence (X). The biotinylated tag is indicated by the blue box. In the lower panels cytoplasmic extracts from these cells were recovered on streptavidin beads and analyzed for biotinylated proteins by Western blotting with Alexafluor 800 coupled streptavidin, and for Nck1 with anti-HA antibody. The GMP-binding activity of recovered protein (guanylylation) was assayed by incubation with α-[32P]GTP. (TIF) [file pbio.1001933.s004.tif]

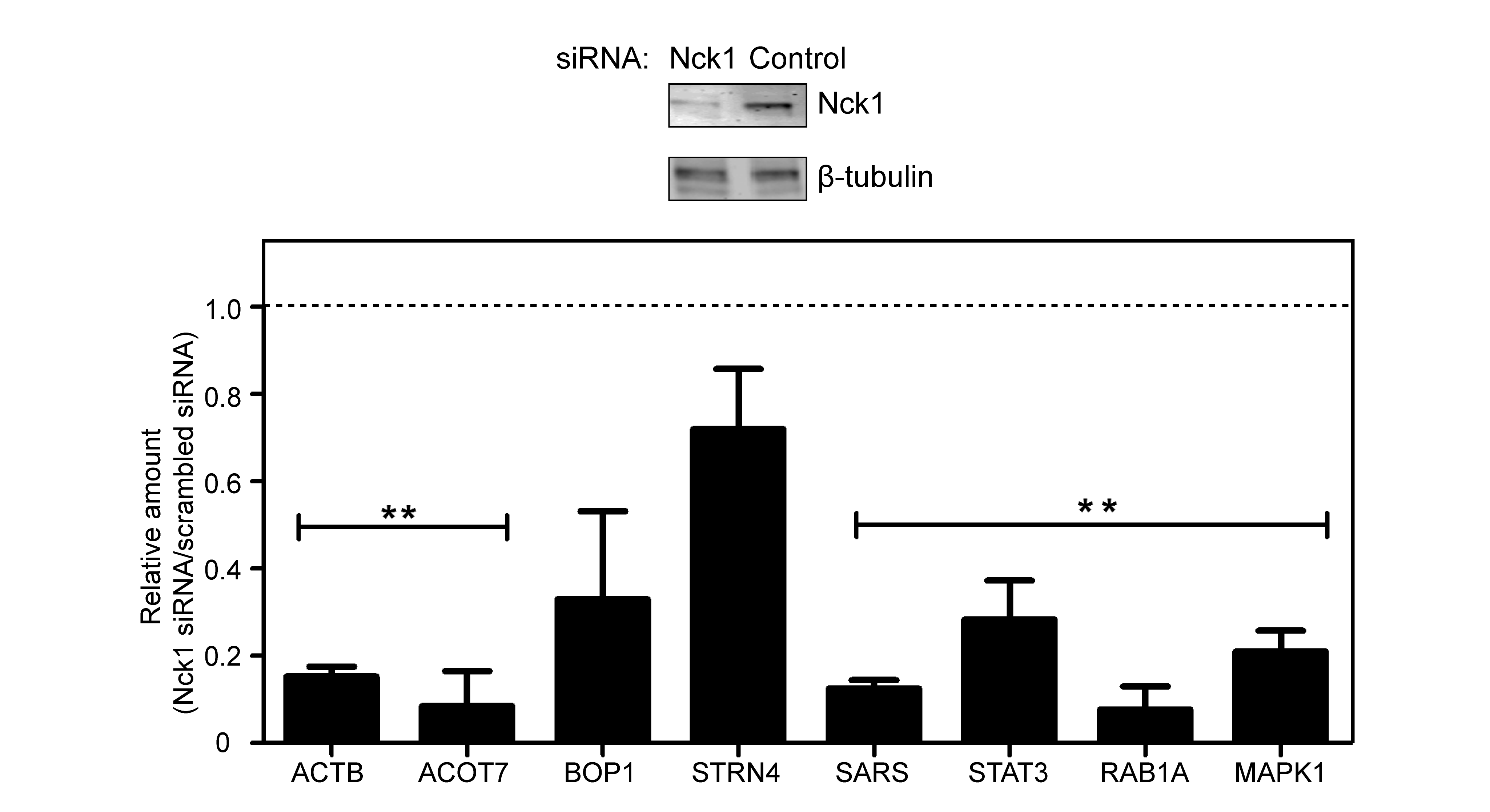

Supplement: Figure S5 — Impact of Nck1 knockdown on steady-state levels of select transcripts. Triplicate cultures of U2OS cells were transfected with Nck1 siRNA or a scrambled control (Scr). The effectiveness of the knockdown is shown by Western blot in the upper panel. The indicated transcripts were quantified by qRT-PCR and the data are shown as the relative amount present in Nck1 knockdown cells normalized to that of the scrambled control. The data represent the mean ± standard deviation. **p<0.005 by unpaired two-tailed Student's t test. (TIF) [file pbio.1001933.s005.tif]

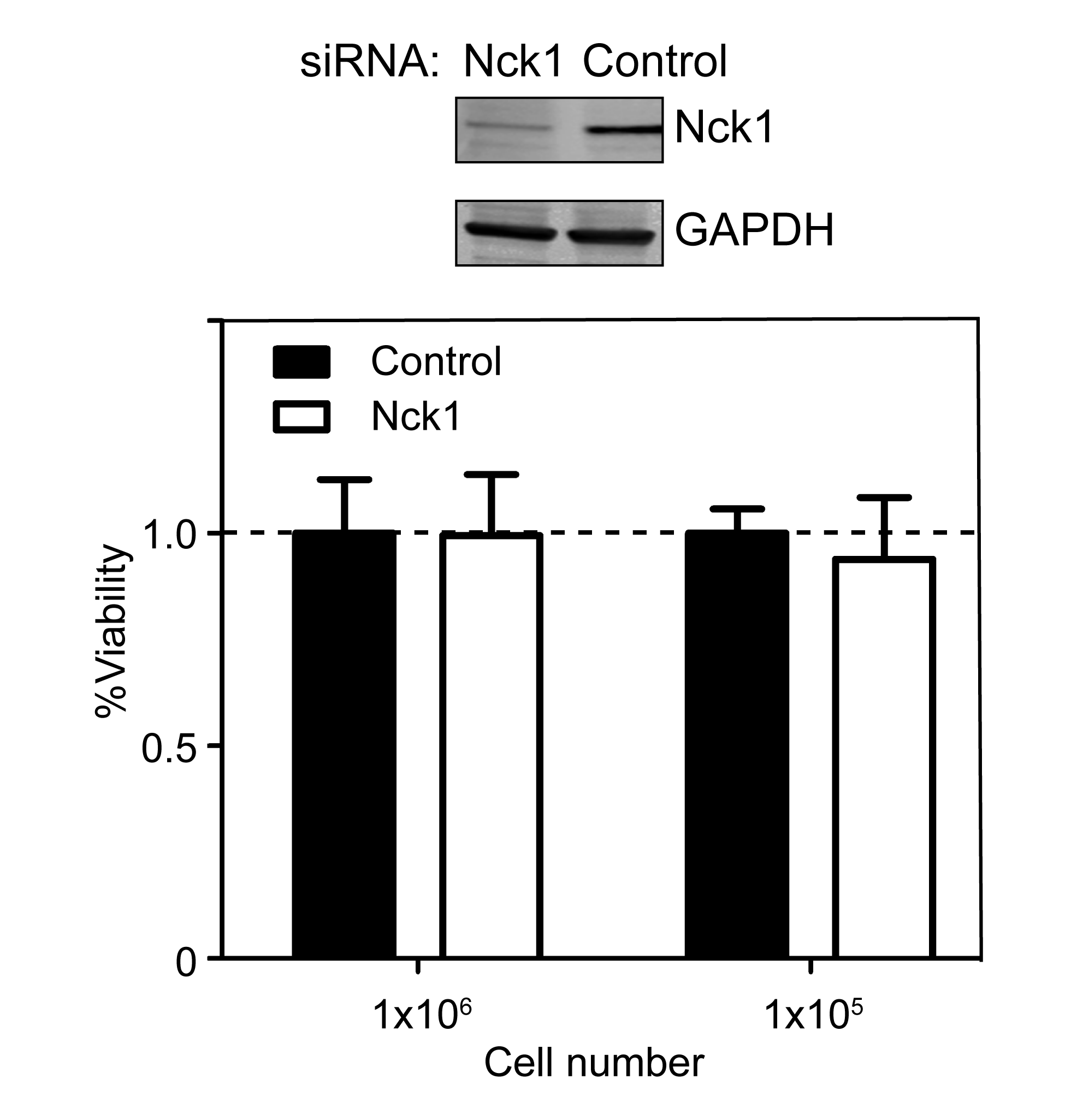

Supplement: Figure S6 — Impact of Nck1 knockdown on cell viability. Triplicate cultures of U2OS cells were transfected with Nck1 siRNA or a scrambled control (Scr). The effectiveness of the knockdown is shown by Western blot in the upper panel. Viability was determined after 72 h by CellTiterGlo assay performed using 1×10^5^ or 1×10^6^ cells. Cells knocked down for Nck1 showed no statistically significant difference in cell viability as determined by two-tailed Student's t test. (TIF) [file pbio.1001933.s006.tif]

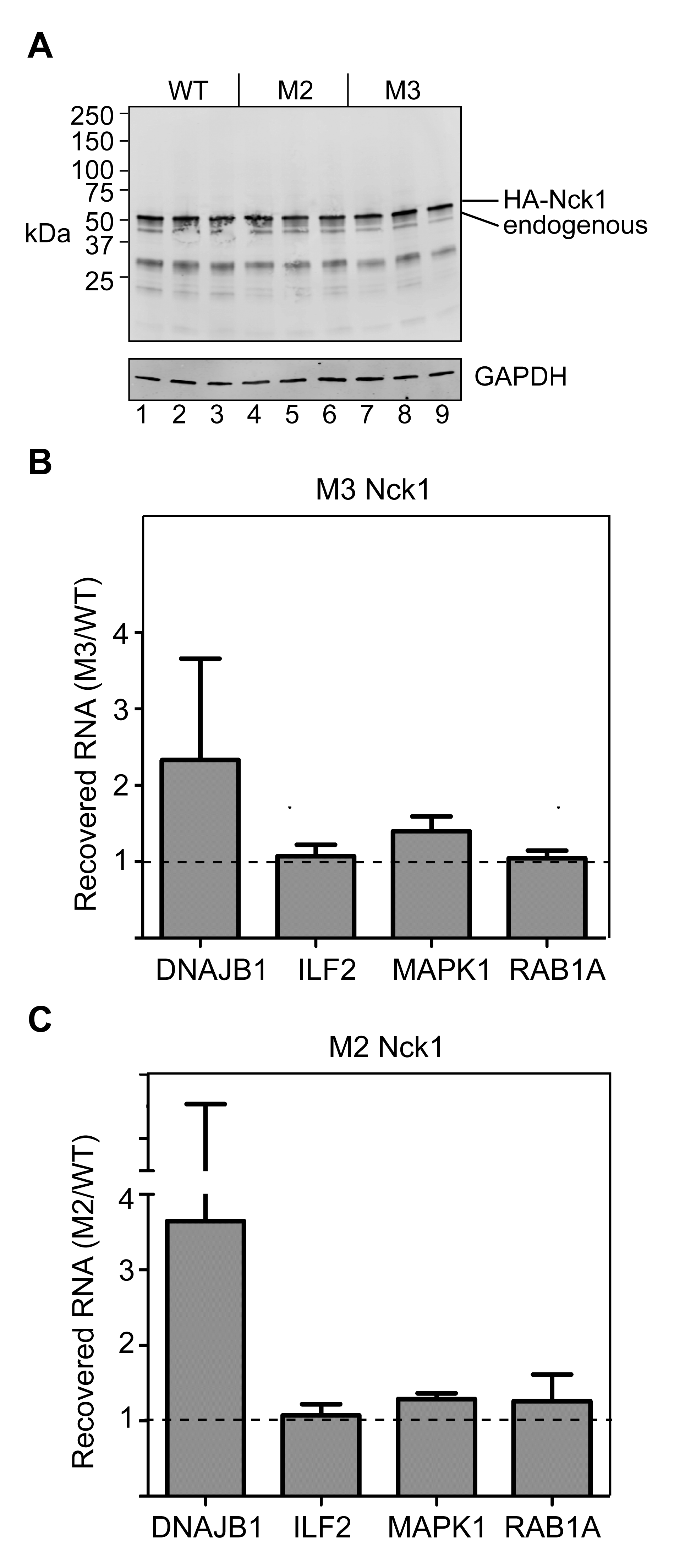

Supplement: Figure S7 — Impact of overexpressing Nck1 mutated in the second and third SH3 domains on steady-state levels of capping inhibited target mRNAs. (A) Triplicate cultures of U2OS cells were transfected with plasmids expressing HA-tagged forms of wild-type Nck1 (WT) or Nck1 with an inactivating mutation in the second SH3 domain (M2) or third SH3 domain (M3). Cytoplasmic extracts from each culture were analyzed by Western blotting with anti-Nck1 antibody (upper panel) or anti-GAPDH (lower panel). (B) The impact of M3 overexpression on DNAJB1, ILF2, MAPK1, and RAB1 mRNA was determined by qRT-PCR performed on cytoplasmic RNA recovered from each of the transfectants in (A). The data are plotted as in Figure 6, with results from M3-expressing cells normalized to results from cells expressing wild-type Nck1. (C) The same analysis of DNAJB1, ILF2, MAPK1, AND RAB1 mRNA was performed on RNA from M2-expressing cells. There was no statistically significant difference between each of the treatments as determined by two-tailed Student's t test. (TIF) [file pbio.1001933.s007.tif]
