# Supplementary material for: The Cytoplasmic Capping Complex Assembles on Adapter Protein Nck1 Bound to the Proline-Rich C-Terminus of Mammalian Capping Enzyme
Source: PLoS Biol. 2014 Aug 19;12(8):e1001933. doi: 10.1371/journal.pbio.1001933 (PMC4138027; doi:10.1371/journal.pbio.1001933)
Supplement: Table S1 — Oligonucleotides and primers used in this study. (DOC) [file pbio.1001933.s008.doc]

| **Primer** | **Sequence** |
| --- | --- |
| YO125 | GAATAGGGCCCTCACTGTCCCTGGGCGGC |
| Tevbio-cCE-F | GCTTGGTACCACCATGGCATCAATGCAGAAGCTG |
| Tevbio-cCE-R | GGTTGGCCGATGCAGTCTTTTG |
| cCE-TevBio-F | CCAAAAGACTGCATCGGCCAACCGAGAATCTGTACTTTCAATCAGAGTT |
| cCE-TevBio-R | AAACGGGCCCTCATTAAACGCCG |
| cCE-F | GCTTGGTACCACCATGGCATCAATGCAGAAGCTG |
| R-Tev Bio | AAACGGGCCCTCATTAAACGCCG |
| Bio FP | GGA GAC CCA AGC TT GGT ACC ATG GAG TTA AAA ACC GCC GC |
| Bio RP | ATT GA TGC CAT GGT GCC AAC GCC GAT CTT GAT TAG |
| cCE-∆ PPP | CCTGACACGGAGCTCATGAAAAGAACTGCATCGGCCA |
| Amp-R | GTCAGAAGTAAGTTGGCCGCAGTGTTATCACTCATGG |
| T7 | TAATACGACTCACTATAGG |
